# Differential Trends in Health Care Utilization and Spending Among Asian American and Pacific Islander Medicare Beneficiaries before and During the COVID-19 Pandemic

**DOI:** 10.1089/heq.2024.0120

**Published:** 2024-12-16

**Authors:** Taylor Melanson, Tanvi Rao, Aditi Pathak, Mike Liu, Tracy Haidar, Rouguia Barry

**Affiliations:** Health Division, American Institutes for Research, Arlington, Virginia, USA.

**Keywords:** health disparities, AAPI, COVID-19, Medicare

## Abstract

**Introduction:**

The COVID-19 pandemic placed unprecedented strains on the U.S. health care system, contributing to significant disruptions of care. COVID-19 was also associated with an increase in negative sentiment toward and hate crimes targeting Asian Americans and Pacific Islanders (AAPI) individuals. The rise in anti-AAPI violence seen across the United States may have discouraged AAPI individuals from seeking medical care beyond the barriers to seeking care imposed on all persons by the pandemic. This study examines how COVID-19 and the concurrent increase in hate crimes targeting AAPI individuals impacted care utilization.

**Materials and Methods:**

We use fee-for-service claims from Medicare beneficiaries enrolled in Parts A and B for 2017–2021. We present descriptive results and use a difference-in-differences-style regression framework to estimate changes in ambulatory utilization associated with the COVID-19 pandemic and compare results across racial/ethnic groups.

**Results:**

The start of the pandemic is associated with decreases in the percentage of beneficiaries with ≥1 ambulatory visit, ambulatory visit rate, and ambulatory spending, among all racial/ethnic groups. AAPI beneficiaries suffer larger disruptions to all three measures of utilization, compared with other racial/ethnic groups.

**Discussion:**

Trends among AAPI beneficiaries are unlike those seen in Black, Hispanic, or White beneficiaries, suggesting that AAPI beneficiaries experience care disruptions different in cause and/or magnitude from the disruptions affecting other groups.

**Conclusions:**

Racial/ethnic disparities may be overlooked if results are only reported for some sub-groups. The experience of AAPI individuals during the COVID-19 pandemic is markedly different from that of other groups and warrants additional study.

## Introduction

The COVID-19 pandemic placed unprecedented strains on the U.S. health care system, contributing to significant disruptions of care, including missed follow-up care for patients, decreased medication adherence,^1^ delayed appointments,^2^ and intentional avoidance or delaying of care.^3^ While health care systems were strained across the country, COVID-19 had a disproportionately large impact on certain demographic groups. Black, Indigenous, and People of Color persons in lower socioeconomic status households were over-represented in many essential industries that remained open during the pandemic.^4,5^

COVID-19 was also associated with an increase in negative sentiment toward and hate crimes targeting Asian Americans and Pacific Islanders (AAPI) individuals.^6,7^ The rise in anti-AAPI violence seen across the United States may have discouraged AAPI individuals from seeking medical care beyond the barriers to seeking care imposed on all persons by the pandemic. Health equity is only achievable when all individuals are capable of attaining their potential well-being^8^ and anti-AAPI violence is a serious obstacle to reaching this goal.

Numerous studies have examined racial disparities in COVID-19-related outcomes but very often they only compare White individuals with Black and/or Hispanic individuals.^9^ Studies that further disaggregate their data often find that AAPI individuals experience notably different outcomes than Black and Hispanic individuals and may or may not experience similar outcomes to White individuals. This has been found when examining rates of positive COVID-19 tests, COVID-19 related hospitalization and death,^5,9,10^ but there are few studies that examine care utilization. For these reasons, we believe that it is essential for researchers to disaggregate their data to the degree possible, not only to understand how different populations may be accessing health care but also to understand potentially heterogenous effects of health care policies and programs.

We examine trends in the use of ambulatory care^1^ among Medicare fee-for-service (FFS) beneficiaries before and after the start of the COVID-19 pandemic and how those trends differ across racial/ethnic groups. Specifically, we are interested in examining how COVID-19 may be associated with differential ambulatory care utilization patterns among AAPI beneficiaries, compared with other beneficiaries.

## Materials and Methods

This analysis uses FFS claim data from a 5-percent simple random sample of Medicare beneficiaries who were enrolled in Medicare Parts A and B between 2017 and 2021. We divide our study period into pre-pandemic (2017 Quarter [Q] 1–2019 Q4) and pandemic (2020 Q1-2021 Q4) based on when the United States declared a public health emergency (PHE). We examine trends in ambulatory care utilization between 2017 Q1 and 2021 Q4, across racial/ethnic groups at the quarter level. We also use a difference-in-differences (DID) style regression framework^2^ at the beneficiary-quarter level to estimate changes in utilization pre- and post-pandemic, for AAPI beneficiaries compared with non-AAPI beneficiaries. This study was deemed exempt by the American Institutes for Research Institutional Review Board.

## Results

### Descriptive results

AAPI beneficiaries in our sample tended to be older than Black and Hispanic beneficiaries but had a similar age distribution to White beneficiaries (Table 1). AAPI beneficiaries were more likely than other beneficiaries to be female and dual eligible but the least likely group to live in rural areas (only 4% rural). White beneficiaries had the lowest prevalence of diabetes, while AAPI beneficiaries had the lowest prevalence of Chronic obstructive pulmonary disease (COPD) and heart failure. All co-morbidities examined declined in the pandemic period, which is consistent with previous findings that COVID-19 was more likely to be fatal for individuals with pre-existing conditions.^11^ AAPI beneficiaries had the lowest rate of ambulatory visits in both the pre-pandemic and pandemic periods. The rate of telehealth utilization in AAPI beneficiaries went from the lowest of all racial/ethnic groups in the pre-pandemic period (0.04%) to the highest of all racial/ethnic groups in the pandemic period (10%). Ambulatory spending was also lower among AAPI beneficiaries than among other beneficiaries, both prior to, and during the pandemic period.

**Table 1.** Pre-Pandemic (2017 Q1–2019 Q4) Population Characteristics by Race/Ethnicity

	Full sample		White beneficiaries		Black beneficiaries		Hispanic beneficiaries		AAPI beneficiaries	
	*N*	%	*N*	%	*N*	%	*N*	%	*N*	%
*N*	15,481,730		13,070,331		1,137,395		831,416		442,588	
Age 65 to <75	8,630,276	55.74	6,884,391	52.67	668,647	58.79	473,674	56.97	232,281	52.48
Age 75 to < 85	5,033,303	32.51	4,224,837	32.32	330,576	29.06	253,290	30.46	144,585	32.67
Age ≥85	2,294,657	14.82	1,961,103	15.00	138,172	12.15	104,452	12.56	65,722	14.85
Male	7,012,248	45.29	5,723,892	43.79	469,489	41.28	372,284	44.78	180,263	40.73
Female	8,945,988	57.78	7,346,439	56.21	667,906	58.72	459,132	55.22	262,325	59.27
Rural	3,384,574	21.86	3,022,644	23.13	156,729	13.78	87,979	10.58	16,820	3.80
Dual eligible	1,870,157	12.08	1,050,862	8.04	287,417	25.27	299,660	36.04	170,868	38.61
Hypertension	9,811,364	63.37	7,959,646	60.90	824,191	72.46	502,597	60.45	270,250	61.06
Diabetes	4,398,447	28.41	3,290,961	25.18	469,853	41.31	334,247	40.20	169,045	38.19
Asthma	755,914	4.88	598,407	4.58	68,179	5.99	45,503	5.47	21,719	4.91
COPD	1,833,109	11.84	1,568,560	12.00	125,248	11.01	73,542	8.85	31,464	7.11
Heart failure	2,318,080	14.97	1,890,431	14.46	210,726	18.53	121,136	14.57	49,870	11.27
Beneficiary-quarters with no ambulatory visit	3,883,987	25.09	3,028,447	23.17	306,120	26.91	282,869	34.02	131,203	29.64
Number of ambulatory visits	67,608,067	n/a	55,632,059	n/a	5,237,468	n/a	3,290,295	n/a	1,724,786	n/a
Telehealth visits	48,742	n/a	40,271	n/a	4,026	n/a	2,468	n/a	700	n/a
Ambulatory visits per 1000 Beneficiary-quarters	4,236.50	n/a	4,256.40	n/a	4,605.40	n/a	3,957.50	n/a	3,896.40	n/a
Beneficiary-quarters with no visits, per 1000 beneficiary-quarters	243.3	n/a	231.7	n/a	269.2	n/a	340.2	n/a	296.4	n/a
Telehealth visits per 1000 beneficiary-quarters	3.1	n/a	3.1	n/a	3.6	n/a	3	n/a	1.6	n/a
Percent of beneficiary-quarters with a telehealth visit	0.10%	n/a	0.10%	n/a	0.10%	n/a	0.10%	n/a	0.00%	n/a
Average ambulatory spending per beneficiary-quarter	$370.80	n/a	$368.65	n/a	$427.11	n/a	$368.30	n/a	$357.39	n/a

AAPI, Asian Americans and Pacific Islanders.

For AAPI beneficiaries, the percentage with at least one ambulatory visit in a quarter decreased 14.7 percentage points between the quarter preceding the PHE (2019 Q4, 70.6%) and the first quarter of the PHE (2020 Q2, 55.9%), while White, Black, and Hispanic, beneficiaries experienced smaller decreases: 9.8 percentage points (77.6% to 67.8%), 8.5 percentage points (73% to 64.5%), and 9.8 percentage points (65.4% to 55.9%), respectively.

For AAPI beneficiaries, the ambulatory visit rate per quarter decreased by 35.7% between the quarter preceding the PHE (3.95) and the first quarter of the PHE (2.54), while White, Black, and Hispanic beneficiaries experienced smaller decreases: 26.5% (4.38 to 3.22), 18.9% (4.63 to 3.76), and 21.7% (3.99 to 3.12), respectively.

For AAPI beneficiaries, ambulatory spending^3^ per quarter decreased by 31.6% between the quarter preceding the PHE ($359.83) and the first quarter of the PHE ($246.94), while White, Black, and Hispanic beneficiaries experienced smaller decreases: 26.2% ($380.63 to $284.13), 16.8% ($432.63 to $360.70), and 19.8% ($374.92 to $302.07), respectively.

Table 1 shows the characteristics of the pre-pandemic population. The pandemic population characteristics were largely similar with the following exceptions: rates of co-morbidities and ambulatory visits decreased in all groups, the rate of telehealth visits increased in all groups, and average ambulatory spending increased in all groups other than AAPI beneficiaries (AAPI beneficiaries had a decrease in average ambulatory spending of $14 per beneficiary-quarter).

### DID results

Before COVID-19, AAPI beneficiaries had a 6% lower likelihood of having at least one ambulatory visit in a quarter, compared with non-AAPI beneficiaries (Table 2). During the pandemic, non-AAPI beneficiaries saw a decrease of 1.1% in the likelihood of having at least one ambulatory visit in a quarter. During the same period, the decrease among AAPI beneficiaries was 3.5% (a relative decline of 2.4 percentage points compared with non-AAPI beneficiaries).

**Table 2.** Beneficiary-Quarter Level Difference-In-Difference Analyses Estimating the Association Between Ambulatory Utilization Measures, the COVID-19 Pandemic, and Being an AAPI Beneficiary, 2017–2021

	Likelihood of 1 or more ambulatory visits in the quarter	Number of ambulatory visits in the quarter	Total ambulatory spending in the quarter
AAPI	−0.062***	−0.764***	−52.328***
	(0.001)	(0.024)	(2.175)
Post	−0.011***	0.097***	31.874***
	(0.000)	(0.005)	(0.401)
AAPI-Post	−0.024***	−0.320***	−24.779***
	(0.001)	(0.029)	(2.749)
Number of Beneficiary-Quarters	25,816,254	25,816,254	25,816,254
Mean	0.757	4.237	370.8

Robust standard errors in parentheses.

Results controlled for age, sex, rurality, dual eligibility, and ambulatory care sensitive conditions. See Appendix A for full results.

*** *p* < 0.01.

AAPI, Asian Americans and Pacific Islanders.

AAPI beneficiaries have an ambulatory visit rate that is 0.76 visits/quarter lower than other beneficiaries, prior to COVID-19. During the pandemic, non-AAPI beneficiaries saw an increase in their visit rate by 0.10 visits/quarter. During the same period, AAPI beneficiaries experienced a decline of −0.22 visits/quarter (a relative decline of 0.32 visits/quarter compared with non-AAPI beneficiaries).

In terms of ambulatory spending, AAPI beneficiaries average $52/quarter less than other beneficiaries prior to COVID-19 and the pandemic is associated with a widening of this gap. While non-AAPI beneficiaries see spending increase by $32/quarter in the post-period, AAPI beneficiaries only increase spending by $7/quarter (a relative decline of $24.78/quarter compared with non-AAPI beneficiaries).

We examine these same measures among the sub-sample of beneficiaries with at least one ambulatory visit in the quarter as a robustness check and find similar patterns of AAPI beneficiaries receiving fewer visits and spending less than other beneficiaries. All DID results are statistically significant at *p* < 0.01.

## Discussion

All racial/ethnic groups studied have reduced ambulatory care utilization early in the COVID-19 pandemic, followed by increasing utilization approaching pre-COVID-19 trends. Compared with other racial/ethnic groups, AAPI beneficiaries experience greater decreases in all three measures of ambulatory utilization.

AAPI beneficiaries experience a decrease in the likelihood of having at least one ambulatory visit roughly 3.5 times greater than the decrease seen among other beneficiaries (−3.5% vs. −1.1%). While visit rates increase for other beneficiaries, they decrease for AAPI beneficiaries. The size of the AAPI decrease was twice the size of the increase seen among other beneficiaries (−0.22 visits/quarter vs. +0.10 visits/quarter). Compared with other beneficiaries, AAPI beneficiaries had lower spending prior to COVID-19, and the gap in spending increased in the post-period. The fact that AAPI beneficiaries experienced the largest increase in telehealth utilization but still experienced an overall decrease in ambulatory visits suggests that telehealth utilization did not compensate for the decrease in in-person visits seen during the study period.

Taken together, these results support the conclusion that the impacts of the COVID-19 pandemic on ambulatory care utilization are not uniform across racial/ethnic groups and that AAPI beneficiaries are impacted more strongly than other racial/ethnic groups. This is supported by the fact that Black and Hispanic beneficiaries, groups shown to have been disadvantaged in other studies,^9,12^ do not show similar utilization trends to AAPI beneficiaries in our study. It is possible that the differential changes seen in AAPI beneficiaries compared with other racial/ethnic groups may be a result of the rise in anti-AAPI sentiment seen in the United States during the COVID-19 pandemic, though it is beyond the scope of this study to prove the existence of a causal connection.

## Conclusions

Many studies of racial/ethnic inequality fail to examine AAPI individuals as a distinct group^9,13,14^ and this is likely obscuring ways in which AAPI individuals may receive different care and/or experience different health outcomes than other racial/ethnic groups. The fact that we see not only inequality between AAPI and White beneficiaries but also differential patterns of change in AAPI beneficiaries relative to Black and Hispanic beneficiaries supports the conclusion that AAPI individuals were negatively impacted by the COVID-19 pandemic in ways that differ from other disadvantaged groups. While AAPI individuals may not have experienced disproportionate rates of COVID-19 incidence,^15^ it is important to understand the ways in which the pandemic differentially impacted the care received by AAPI individuals. We also saw that AAPI beneficiaries had a greater increase in telehealth utilization than other groups, suggesting that telehealth may be a valuable tool to improve access to care among vulnerable or disadvantaged groups. As the population of AAPI individuals in the United States continues to grow, it is vitally important that researchers consider and examine the ways in which this group may differ from other racial/ethnic groups to ensure efforts to increase health equity do not leave anyone behind.

## Notes

^1^
Ambulatory care is used throughout to refer to services provided in hospital outpatient settings or by professional providers captured in Carrier claim files. This includes telehealth services.

^2^
γ=β0+β1(Post)+β2(AAPI)+β3(Post∗AAPI)+β4(Controls)+ϵwhere y = measure of utilization; Post = 1 (from 2020 Q1 to 2021 Q4), else = 0; AAPI = 1 if the beneficiary is an Asian American/Pacific Islander, else = 0; Post*AAPI = 1 if Post = 1 and AAPI = 1, else = 0; Controls represents the vector of control variables included but not shown in figures.

^3^
Spending is defined as “payment due to the provider,” the sum of Medicare, Beneficiary, and Primary Payer payments. See https://www2.ccwdata.org/documents/10280/19002248/ccw-technical-guidance-getting-started-with-cms-medicare-administrative-research-files.pdf, page 37.

## Abbreviations Used

AAPI: Asian Americans and Pacific Islanders
COPD: Chronic obstructive pulmonary disease
DID: difference-in-differences
FFS: fee-for-service
PHE: public health emergency
